# Catecholaminergic Innervation of Periventricular Neurogenic Regions of the Developing Mouse Brain

**DOI:** 10.3389/fnana.2020.558435

**Published:** 2020-09-23

**Authors:** Mareike Fauser, Grit Weselek, Christine Hauptmann, Franz Markert, Manfred Gerlach, Andreas Hermann, Alexander Storch

**Affiliations:** ^1^Department of Neurology, University of Rostock, Rostock, Germany; ^2^Division of Neurodegenerative Diseases, Department of Neurology, Technische Universität Dresden, Dresden, Germany; ^3^German Centre for Neurodegenerative Diseases (DZNE), Bonn, Germany; ^4^Clinic for Child and Adolescent Psychiatry, Psychosomatics and Psychotherapy, Center for Mental Health, University Hospital Würzburg, Würzburg, Germany; ^5^Translational Neurodegeneration Section “Albrecht-Kossel”, Department of Neurology, University of Rostock, Rostock, Germany

**Keywords:** brain development, ventricular zone, catecholamines, norepinephrine, dopamine, neurogenesis

## Abstract

The major catecholamines—dopamine (DA) and norepinephrine (NE)—are not only involved in synaptic communication but also act as important trophic factors and might ultimately be involved in mammalian brain development. The catecholaminergic innervation of neurogenic regions of the developing brain and its putative relationship to neurogenesis is thus of pivotal interest. We here determined DA and NE innervation around the ventricular/subventricular zone (VZ/SVZ) bordering the whole ventricular system of the developing mouse brain from embryonic day 14.5 (E14.5), E16.5, and E19.5 until postnatal day zero (P0) by histological evaluation and HPLC with electrochemical detection. We correlated these data with the proliferation capacity of the respective regions by quantification of MCM2^+^ cells. During development, VZ/SVZ catecholamine levels dramatically increased between E16.5 and P0 with DA levels increasing in forebrain VZ/SVZ bordering the lateral ventricles and NE levels raising in midbrain/hindbrain VZ/SVZ bordering the third ventricle, the aqueduct, and the fourth ventricle. Conversely, proliferating MCM2^+^ cell counts dropped between E16.5 and E19.5 with a special focus on all VZ/SVZs outside the lateral ventricles. We detected an inverse strong negative correlation of the proliferation capacity in the periventricular neurogenic regions (log-transformed MCM2^+^ cell counts) with their NE levels (*r* = −0.932; *p* < 0.001), but not their DA levels (*r* = 0.440; *p* = 0.051) suggesting putative inhibitory effects of NE on cell proliferation within the periventricular regions during mouse brain development. Our data provide the first framework for further demandable studies on the functional importance of catecholamines, particularly NE, in regulating neural stem/progenitor cell proliferation and differentiation during mammalian brain development.

## Introduction

There is a multitude of research exploring the distribution of the major catecholamines—namely dopamine (DA) and norepinephrine (NE, also called noradrenaline)—in the mammalian brain including mammalian brain development. Noradrenergic neurons in the Locus coeruleus (LC) as the major noradrenergic formation in the mammalian brain are among the earliest born neurons followed by the dopaminergic system in later embryonic stages (Coyle, 1977; Steindler and Trosko, 1989; Pattyn et al., 2000; Aroca et al., 2006). LC neurons widely spread all over the brain with the striatum as one major exception (Foote et al., 1983; Erdtsieck-Ernste et al., 1991; Aston-Jones et al., 1995). Although the LC noradrenergic neurons are born early in development at around murine embryonic day 9 (E9)–E11 (all embryonic stages in this article are adapted to mouse embryonic ages post-fertilization through Carnegie stage comparisons; Lauder and Bloom, 1974; Coyle, 1977; Coyle and Molliver, 1977), the ascending noradrenergic projections throughout the brain needs several more days and reach the diencephalon at E13–E14 and cortical regions at E16–E17 with an increase in NE content as measured by HPLC of various brain areas starting around E15 to E17 in rodents depending on the brain region (Olson and Seiger, 1972; Lauder and Bloom, 1974; Coyle, 1977; Kohno et al., 1982; Berger and Verney, 1984; Murrin et al., 2007). The cortical distribution and density of NE fibers achieve their final pattern at postnatal day 7 (P7) and the adult extent in the 3rd to 4th postnatal week (Berger and Verney, 1984; Murrin et al., 2007). Similarly, tyrosine hydroxylase (TH) immunoreactivity in the ventral midbrain is detected from E11 to E12, and DA immunoreactivity is found from E12 (Olson and Seiger, 1972; Lauder and Bloom, 1974; Tomasini et al., 1997). This again contrasts with the later increase in DA tissue content from E13 to E15 (Ribary et al., 1986; Tomasini et al., 1997). The reported time offset between cellular and histological development of catecholaminergic systems and their subsequent functional development urgently necessitates the additive measurement of tissue levels of catecholaminergic neurotransmitters. Together, the development of both major catecholaminergic systems of the mammalian brain starts at a similar embryonic (postconceptional) age, is well pronounced during late embryonic development, and become functional just before birth. However, the available studies on catecholaminergic systems development strongly focused on neurotransmitter function within the nigrostriatal and mesolimbic dopaminergic system and the LC noradrenergic system innervating the cerebral cortex.

Catecholamines are not only involved in synaptic communication but also act as important trophic factors (Felten et al., 1982; Gustafson and Moore, 1987; Berger-Sweeney and Hohmann, 1997). In this respect, NE is considered to play a trophic role in brain maturation particularly cortical dendritic structuring (Felten et al., 1982; Gustafson and Moore, 1987; Berger-Sweeney and Hohmann, 1997). However, most studies used mechanical or toxic lesions of the NE system during the early postnatal period and only addressed cortical development (for review see Berger-Sweeney and Hohmann, 1997). The one study using prenatal lesioning of the NE system by intrauterine 6-hydroxydopamine application at E17 revealed no major alterations in cortical morphology in adulthood but did not study other brain regions (Lidov and Molliver, 1982). The more recent approach to disrupt NE innervation by knocking-out the gene encoding for dopamine-β-hydroxylase (DbH), the rate-limiting enzyme of NE production, showed that NE is essential for rodent fetal development with almost 100% mortality within the first days of life (Thomas et al., 1995). The brain morphology of these animals has however not been reported in detail yet. Substitution of NE by L-3, 4-dihydroxyphenylserine (DOPS) bypassing DbH restored NE synthesis and completely rescued the survival of DbH knock-out animals. However, in these mice, the postnatal development of the cerebellum and the noradrenergic systems was independent of NE (Jin et al., 2004). The reason for the prenatal mortality of DbH knock-out mice remains unclear but is most likely due to cardiovascular instability, which for unknown reasons stabilizes shortly after birth allowing the withdrawal of DOPS supplementation. Whether this instability arises from central or peripheral pathology remains enigmatic. The lethal phenotype without indirect NE supplementation during crucial developmental stages makes it impossible to accurately study embryonic/fetal brain development under NE depletion in this model. These data together with the recently described role of norepinephrine as a negative regulator of neural stem cell proliferation in the adult brain (Weselek et al., 2020) raised the question of whether catecholamines show analogous trophic actions during embryonic brain development beyond cortical development. We therefore comparatively determined the catecholaminergic innervation and the neural stem/progenitor cell proliferation capacity of the ventricular zone/subventricular zone (VZ/SVZ) bordering the whole ventricular system of the developing mouse brain from E14.5 until postnatal day zero (P0).

## Materials and Methods

### Animals and Tissue Processing

Whole brains were dissected from embryos from timed-pregnant C57BL/6J mice at E14.5, E16.5, E19.5 (histology only), or newborn C57BL/6J mice (P0). All animal protocols were reviewed and approved by Animal Welfare Committee at the Technische Universität Dresden and Landesdirektion Sachsen, Dresden, Germany (governmental authorities). The tissue was fixed for 24 h in 4% paraformaldehyde (Merck, Darmstadt, Germany) and kept in 30% sucrose (Carl Roth, Karlsruhe, Germany) in PBS (Thermo Fisher Scientific, Waltham, MA, USA). Then brains were snap-frozen, coronal sections at 20 μm thickness were prepared using a cryotome (Leica Biosystems, Nussloch, Germany) and mounted on Superfrost Plus slides (Thermo Fisher Scientific, Waltham, MA, USA). The slides were stored at 4°C until staining. A total of three animals per group were examined for each experiment; all data were gathered from randomly chosen fetuses from three independent litters.

### Immunohistochemistry

Cryosections were pre-incubated in 3% blocking donkey serum (Jackson Immunoresearch, West Grove, IA, USA) containing 0.2% Triton X-100 (Thermo Fisher Scientific, Waltham, MA, USA) in PBS for 2 h at room temperature and then incubated overnight at 4°C with primary antibodies followed by secondary fluorescence conjugated antibodies for 1 h at room temperature. Cell nuclei were counterstained with 4,6-diamidino-2-phenylindole (DAPI, Thermo Fisher Scientific, Waltham, MA, USA). The following primary antibodies were used: sheep or rabbit anti-TH 1:500 (Pel-Freez, Rogers, Arkansas, RRID:AB_461070 and RRID:AB_461064); mouse anti-Nestin 1:500 (Chemicon, Thermo Fisher Scientific, RRID:AB_2251134); rat anti-DAT 1:500 (Merck, RRID:AB_2190413); rabbit anti-MCM2 1:500 (Abcam, Cambridge, UK, RRID:AB_881276; mouse anti-NET 1:200 (MAB Technologies, Neenah, WI, USA, RRID:AB_2571639). Fluorescence labeled secondary antibodies were purchased from (Molecular Probes, Thermo Fisher Scientific, Waltham, MA, USA). Images were captured using a fluorescence microscope (Leica DM IRE2, Wetzlar, Germany) or a Zeiss confocal microscope (LSM 700; Zeiss, Oberkochen, Germany) equipped with DAPI/UV, krypton, krypton/argon, and helium lasers.

### HPLC Assay of Tissue Catecholamine Content

Tissue from the same periventricular regions (200 μm surrounding the ventricles) as those used for immunohistochemistry was microdissected from mouse brains at E14.5, E16.5, P0 and adult (8–12 weeks of age, male). For this purpose, the brains were removed from out of the skull and sliced into coronal sections of approximately 1 mm which were kept on ice or a cooling plate until further preparation. With the help of a dissection microscope (magnification: ×100) approximately 200 μm tissue surrounding the ventricles at the appropriate zone was removed by one experienced experimenter (Grit Weselek) and the pieces were snap-frozen in liquid nitrogen. The 200 μm were chosen to secure enough material for HPLC analysis while including the major part of VZ/SVZ. For further HPLC analysis, the tissue samples were thawed, weighed, and sonicated in 0.05 M perchloric acid for 60 s resulting in 10% (w/v) homogenate suspensions. The mean ± s.e.m. weights of tissue samples of 22 ± 2 (range: 9–45) mg per tissue sample were in the typical range of previous studies (Wagner et al., 1982) and did not show any significant differences between experimental conditions (two-way ANOVA with VZ regions and developmental stages as fixed factors revealed no significant interaction effect of VZ regions and stages on tissue sample weights (*p* = 0.225, *F*-value = 1.4) and no significant differences among VZ regions (*p* = 0.468, *F*-value = 0.9) and stages (*p* = 0.840, *F*-value = 0.2). These were centrifuged at 48,000× g for 20 min at 4°C, and 50 μl samples of the supernatants injected directly into high-performance liquid chromatography (HPLC) system with electrochemical detection (Gynkotek GmbH, Germering, Germany) for analysis of tissue DA, NE, and epinephrine according to the method described in detail previously (Wagner et al., 1982; Gerlach et al., 1996; Kuhn et al., 1996). The HPLC system consisted of an AGILENT 1100 series (Bio-Rad, Munich, Germany), a Nucleosil 120-5C18 reverse phase (250 × 4.6 mm) analytical column (Macherey-Nagel, Düren, Germany), an electrochemical detector (model 1640; Bio-Rad, Munich, Germany). Detector data were recorded and analyses using an AGILENT Chem Station for LC9D (Bio-Rad). Concentrations were calculated from the peak height with the aid of an external standard. The detection limit of the HPLC system was 0.1 ng/ml (corresponding to 1 ng/g brain tissue) for all catecholamines. The investigator was blind to the experimental condition.

### Cell Counting and Statistical Analysis

For quantification of proliferating mini-chromosome maintenance protein 2 (MCM2)^+^ cells within the ventricular zone/subventricular zone (VZ/SVZ) of the various periventricular regions [lateral wall of the lateral ventricles (LV_lateral_), medial wall of the lateral ventricles (LV_medial_), third ventricle (3V), aqueduct (Aq) and fourth ventricle (4V) according to the Prenatal Mouse Brain Atlas by Schambra (Schambra, 2008)], in any given experiment the number of MCM2^+^ cells was determined in a 130 μm section along the VZ/SVZ of every 12th coronal section using laser scanning confocal microscopy images processed with ImageJ [National Institutes of Health (NIH), Bethesda, MD, USA]. Data are displayed as normalized to 100 μm^2^ VZ/SVZ surface area for each region. Statistical analyses were done with SPSS software, version 21.0 or newer (SPSS, Chicago, IL, USA). We applied various linear and nonlinear regression analyses and logarithm functions nicely matching the MCM2^+^ cell counts and NE levels and rendered a correlation coefficient (*r*^2^) that was 0.858. Curve linearization was thus made by log transformation of results. Correlation analyses to assess the association of stem cell proliferation within the VZ/SVZ with catecholamine levels by using multiple linear regression analyses and Pearson correlation. Comparisons were made, as appropriate, with a two-sample *t*-test, Mann–Whitney *U* test, or χ^2^ test. Two-sided *p*-values of less than 0.05 were deemed statistically significant.

## Results

### Formation of a Rostrocaudal Proliferation Gradient Within the VZ/SVZ During Mouse Brain Development

**Figure 1:**
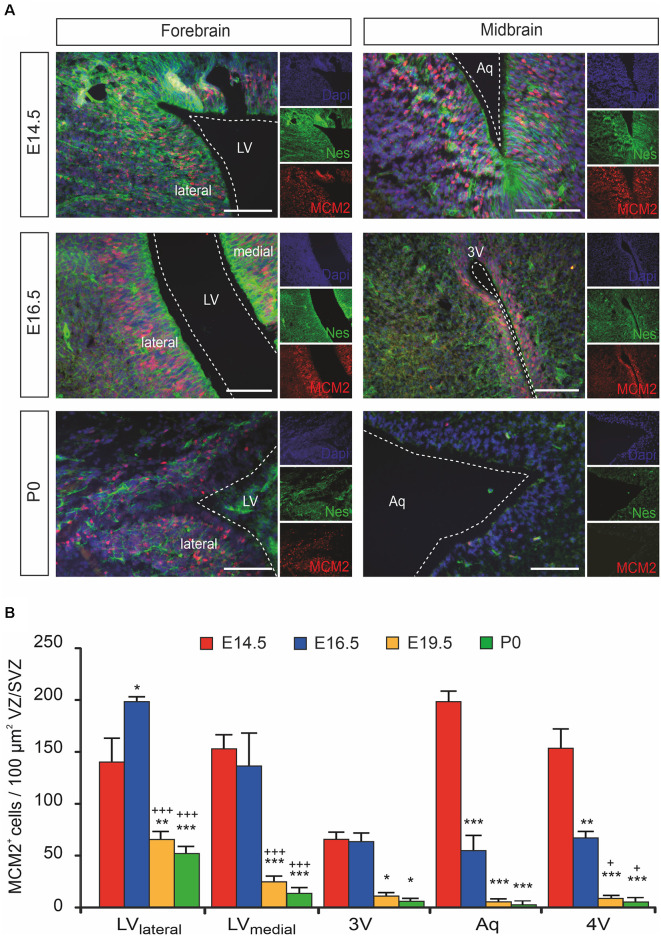
The proliferation of ventricular/subventricular zone (VZ/SVZ) neural stem/progenitor cells (NPCs) within the developing mouse forebrain and midbrain at E14.5, E16.5, E19.5 (quantitative results only), and P0. **(A)** Proliferating stem/progenitor cells were co-stained with MCM2 (red) and Nestin (green) in VZ/SVZ. Cell nuclei are counterstained with 4,6-diamidino-2-phenylindole (DAPI). Scale bar: 50 μm. **(B)** Quantitative immunohistochemistry revealed a dramatic reduction to almost complete loss of proliferating (MCM2^+^) cells during development within the VZ/SVZs except for the lateral VZ/SVZ of the lateral ventricles. Two-way ANOVA with *post hoc* *t-test* and Bonferroni adjustment with VZ/SVZ regions and developmental stages as fixed factors revealed that brain regions and embryonic stages had a significant interaction effect on MCM2^+^ cells per 100 μm^2^ (*P* < 0.001, *F*-value = 5.7) and significant differences among VZ/SVZ regions (*P* < 0.001, *F*-value = 17.1) and embryonic stages (*P* < 0.001, *F*-value = 95.0). *P*-values from *post hoc* tests with Bonferroni adjustment are *P < 0.05, **P < 0.01 and ***P < 0.001 when compared to E14.5 and ^+^*P* < 0.05 and ^+++^*P* < 0.001 when compared to E16.5 (only *P*-values among developmental stages are displayed for clarity; complete statistics in Supplementary Tables S1A,B). 3V, third ventricle; Aq, aqueduct; LV, lateral ventricle; LV_lateral_, lateral wall of the lateral ventricles; LV_medial_, medial wall of the lateral ventricles; MCM2, mini-chromosome maintenance protein 2; Nes, Nestin.

Even though the proliferation capacity of the forebrain proliferative zone (ventricular zone/subventricular zone [VZ/SVZ]) bordering the lateral ventricles on their lateral side (LV_lateral_) during mouse brain development is already described in detail, the time course of proliferation changes within the VZ/SVZ of the medial wall of the LV (LV_medial_) and within the more caudal VZ/SVZ bordering the third ventricle (3V), the aqueduct (Aq) and the fourth ventricle (4V) is not described in detail yet. To determine the proliferation capacity within the VZ/SVZ of the developing mouse brain, we here used the DNA replication licensing factor MCM2 as an established proliferation marker of Nestin^+^ neural stem/progenitor cells (von Bohlen und Halbach, 2011). As shown in Figures 1A,B, quantitative immunohistochemistry revealed a dramatic reduction to almost complete loss of proliferating (MCM2^+^) neural stem/progenitor cells within all proliferative zones between E14.5 and E19.5 except for the VZ/SVZ of LV_lateral_. There were no relevant changes in proliferation patterns between E19.5 and P0. Together, we demonstrated the formation of a rostrocaudal gradient of the VZ/SVZ proliferation capacity during mouse brain development established between E14.5 and E16.5 and persisting until birth with high proliferation capacity in the rostral part (proliferative zone of the LV_lateral_) down to a low proliferation capacity in the VZ/SVZ surrounding the third-to-fourth ventricle.

### Catecholaminergic Innervation of the VZ/SVZ During Mouse Brain Development

**Figure 2:**
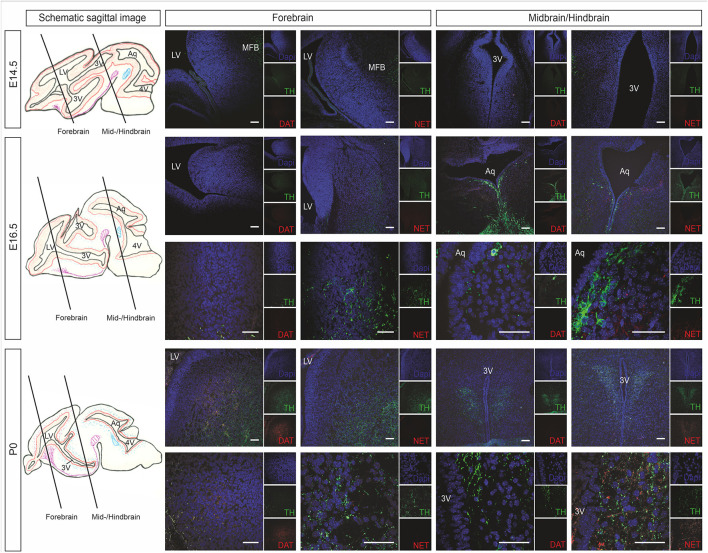
Catecholaminergic innervation of the developing mouse brain (at developmental stages E14.5, E16.5, and P0) determined by immunostaining against the DAT and NET. The left panel shows schematic sagittal brain images illustrating the forebrain and midbrain/hindbrain sections. Representative immunohistological microphotographs of TH in green as a marker for catecholaminergic neurons as well as of DAT as a marker for dopaminergic cells and NET for norepinephrinergic cells. Lower panels in E16.5 and P0 represent individual images of forebrain or midbrain/hindbrain sections in higher magnification. Cell nuclei are counterstained with DAPI. Scale bars: 100 μm (E14.5, upper panels of E16.5 and P0) and 50 μm (lower panels of E16.5 and P0). 3V, third ventricle; Aq, aqueduct; DAT, dopamine transporter; LV, lateral ventricle; MFB, medial forebrain bundle; NET, norepinephrine transporter; TH, tyrosine hydroxylase.

Catecholaminergic innervation of the developing mouse brain was determined by immunostaining against the common catecholaminergic marker tyrosine hydroxylase (TH), the dopamine transporter (DAT), and norepinephrine transporter (NET) at E14.5, E16.5, and P0. Figure 2 shows representative immunohistological microphotographs of TH as well as of DAT and NET staining during mouse brain development. Histology data demonstrated an increase of TH^+^ fibers in all VZ/SVZ and adjacent regions during development after E14.5, but differential patterns of DAT^+^ and NET^+^ fibers: While DAT^+^ innervation increased between E16.5 and P0 within the striatum and the adjacent proliferative zone of the LV_lateral_, we did not detect relevant changes in striatal NET^+^ fibers in the respective region. In all other VZ/SVZ regions, we detected no relevant changes in DAT^+^ fiber density during brain development (Figure 2). In contrast, NET^+^ fiber intensity displayed an increase during development after E14.5 in brain tissue surrounding the caudal VZ/SVZ regions of the 3V and Aq.

**Figure 3:**
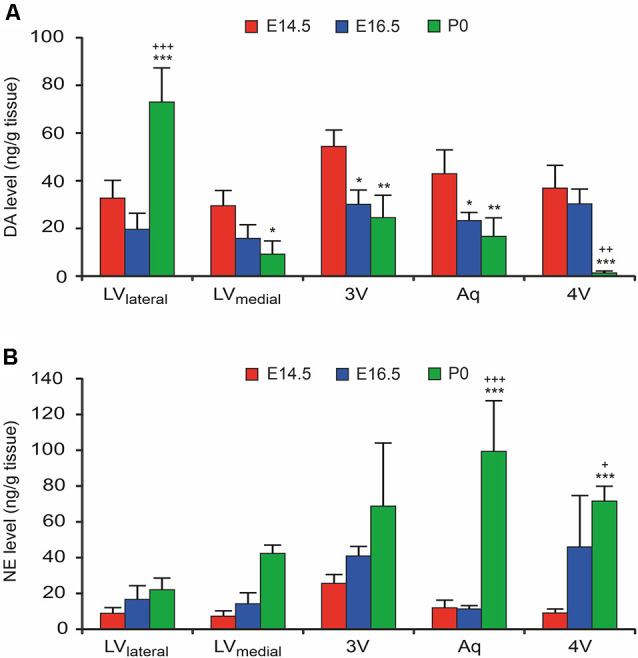
Catecholamine levels in microdissected VZ/SVZ regions of the developing mouse brain (at developmental stages E14.5, E16.5. and P0). Catecholamine levels were measured in microdissected VZ/SVZ regions using an HPLC with electrochemical detection and normalized to tissue weight. **(A)** Dopamine (DA) levels were increasing in the VZ/SVZ of the LV lateral walls (LV_lateral_), while they were continuously declining during development in the caudal VZ/SVZ regions. Two-way ANOVA with brain regions and developmental stages as fixed factors revealed that VZ/SVZ regions and stages had a significant interaction effect on DA levels (*P* < 0.001, *F*-value = 13.3) and significant differences among VZ/SVZ regions (*P* < 0.001, *F*-value = 10.3) and stages (*P* < 0.001, *F*-value = 14.1). **(B)** In contrast to DA, norepinephrine (NE) showed continuously increasing levels in the caudal (hindbrain) VZ/SVZ regions during development. Two-way ANOVA revealed that VZ/SVZ regions and stages had a significant interaction effect on NE levels (*P* = 0.001, *F*-value = 4.3) and significant differences among VZ/SVZ regions (*P* = 0.003, *F*-value = 5.3) and stages (*P* < 0.001, *F*-value = 32.4). *P*-values from *post hoc* tests with Bonferroni adjustment are *P < 0.05, **P < 0.01 and ***P < 0.001 when compared to E14.5 and ^+^*P* < 0.05, ^++^*P* < 0.01 and ^+++^*P* < 0.001 when compared to E16.5 (only *P*-values among developmental stages are displayed for clarity; complete statistics in Supplementary Tables S2, S3). 3V, third ventricle; 4V, fourth ventricle; Aq, aqueduct; LV_lateral_, lateral wall of the lateral ventricles; LV_medial_, medial wall of the lateral ventricles.

To confirm the functional catecholaminergic innervation of the VZ/SVZ, i.e., the actual catecholamine releases, we measured the levels of DA, NE, and epinephrine in microdissected proliferative zones periventricular regions containing the VZ/SVZ of the developing mouse brain at E14.5, E16.5, and P0 using an HPLC-electrochemical detection-based method. Since the thickness of the VZ/SVZ areas varies within the developing brain and changes during the developmental process and may thus not exactly correspond to the analyzed tissue samples, the preparation method used does not fully rule out partial contamination of HPLC samples with periventricular regions outside the VZ/SVZ. Nevertheless, in agreement with the DAT histology data, DA showed increased levels in the VZ/SVZ of the LV_lateral_ at P0, while there was a continuous decline during development in the caudal VZ/SVZ regions (Figure 3A, for statistics, refer to Supplementary Table S2). In contrast, in correspondence with the NET staining, NE levels showed continuously increasing levels in the caudal (hindbrain) VZ/SVZ regions during development between E16.5 and P0 (Figure 3B, Supplementary Table S3). The pattern of catecholaminergic innervation was similar at P0 compared to the adult, even though the catecholamine levels in the adult brain were much higher compared to the developing brain (Supplementary Figure S1). We did not detect epinephrine/adrenaline (<1 ng/g brain tissue) in any VZ/SVZ region of the developing and adult mouse brain.

### Correlations Between Catecholaminergic Innervation and Stem Cell Proliferation

**Figure 4:**
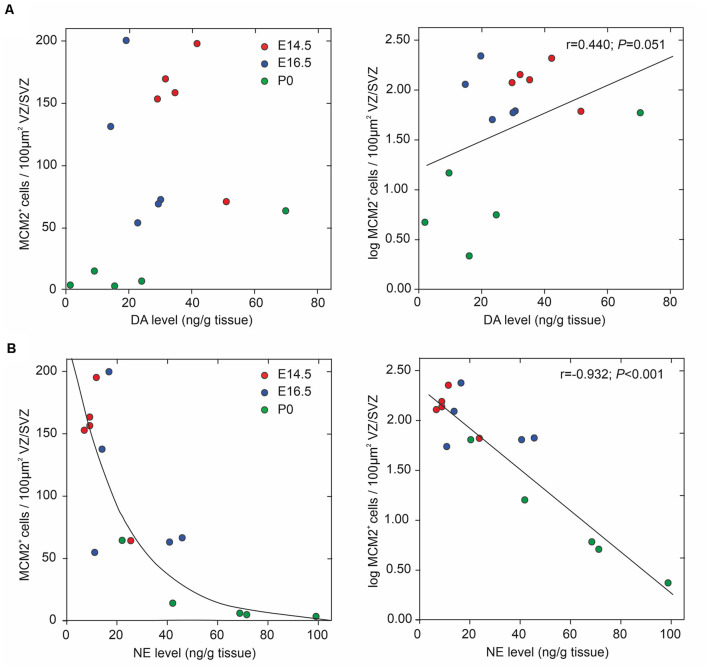
Correlation between periventricular catecholamine levels and neural stem/progenitor cells (NPC) proliferation in VZ/SVZ regions (at developmental stages E14.5, E16.5, and P0). NPC proliferation rate was estimated by quantitative MCM2 immunohistochemistry and DA levels **(A)** and NE levels **(B)**, respectively, were measured in microdissected VZ/SVZ regions using HPLC with electrochemical detection. Pearson correlation tests revealed correlations with a magnitude greater than |0.5| of log-transformed MCM2^+^ cell counts with NE levels, but not with DA levels (*r* = 0.440; *p* = 0.051). The Pearson correlation coefficients for the respective variables are displayed in the upper right part of the graphs. Multiple linear regression model analysis revealed that log-transformed MCM2^+^ cell counts were significantly associated only with NE levels (corrected *r^2^* = 0.858, *β* = −0.701, *p* = 0.003), but not with DA levels (*β* = 0.045, *p* = 0.694), embryonic stage (*β* = −0.264, *p* = 0.137) or brain region (*β* = −0.053, *p* = 0.674).

We assessed potential associations of the periventricular proliferative capacity of MCM2^+^ neural stem/progenitor cells within the VZ/SVZ with periventricular DA and NE levels, developmental stages, and periventricular brain regions (Figure 4). Pearson correlation tests revealed correlations with a magnitude greater than |0.5| of log-transformed MCM2^+^ cell counts with NE levels (Pearson correlation coefficient *r* = −0.932; *p* < 0.001) and developmental stages (*r* = −0.789; *p* < 0.001). We found no correlations with a magnitude greater than |0.5| of log-transformed MCM2^+^ cell counts with DA levels (*r* = 0.440; *p* = 0.051) and brain regions (*r* = −0.328; *p* = 0.117). Multiple linear regression model analysis revealed that log-transformed MCM2^+^ cell counts were significantly associated only with NE levels (corrected *r^2^* = 0.858, *β* = −0.701, *p* = 0.003), but not with DA levels (*β* = 0.045, *p* = 0.694), developmental stage (*β* = −0.264, *p* = 0.137) or brain region (*β* = −0.053, *p* = 0.674). Hierarchical stepwise multiple regression analysis using sequences of F-tests confirmed that VZ/SVZ log-transformed MCM2^+^ cell counts were significantly correlated with NE levels.

## Discussion

**Figure 5:**
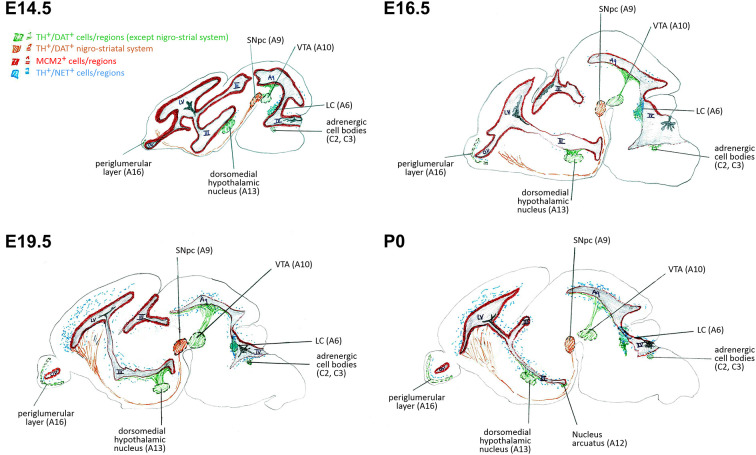
Schematic representation of correlations of neural stem/progenitor cell proliferation and catecholaminergic innervation within VZ/SVZ regions at E14.5, E16.5, E19.5, and P0. III, third ventricle; IV, fourth ventricle; Aq, aqueduct; DAT, dopamine transporter; LC, *locus coeruleus*; LV, lateral ventricle; MCM2, mini-chromosome maintenance protein 2; NET, norepinephrine transporter; OV, olfactory ventricle; SNpc, *substantia nigra pars compacta*; TH, tyrosine hydroxylase; VTA, ventral tegmental area.

We investigated the specific catecholaminergic innervation of the proliferative zones (VZ/SVZ) in the developing mouse brain and its putative association with their respective proliferation capacities throughout the ventricular system. We thereby demonstrate an inverse logarithmic correlation of the proliferation capacity of the VZ/SVZ with the NE content of the corresponding area during brain development, but no correlation with their DA levels. Since the NE neurotransmitter system develops after E14.5 in the midbrain/hindbrain regions bordering the ventricular system and omits the striatum, it is of particular relevance for the caudal VZ/SVZ regions. Figure 5 displays a graphical synopsis summarizing the association of the catecholaminergic neurotransmitter systems development with the proliferation capacity in the VZ/SVZ bordering the ventricular system from its rostral (forebrain LV_lateral_) to the caudal parts within the midbrain/hindbrain (3V through 4V) during mouse brain development. These data imply that NE might be a putative humoral factor involved in the physiological decrease of neural stem/progenitor cell proliferation during prenatal development. Consistently, we recently detected a similar role of NE in the adult brain with inhibitory effects on the proliferative activity of adult neural stem cells in the consecutive neurogenic regions (Weselek et al., 2020). However, the physiological significance of our present findings needs to be determined by functional studies, for example by pharmacologic or genetic manipulation of DA and/or NE levels during the various development stages of the brain.

We used mini-chromosome maintenance protein 2 (MCM2) as a marker for proliferating cells because it is known to be directly involved in the cell cycle process through licensing DNA replication (Masai et al., 2010; Kuipers et al., 2011) and therefore marks proliferating regions (Maslov et al., 2007). Unlike other common markers, such as ph3 or Ki67, MCM2 is required for the initial cell cycling process (Stoeber et al., 2001). This allows the analysis of a broader spectrum of proliferating cells as compared to ph3 and gave a better signal than most Ki67 stainings. Regarding validity, MCM2 shows a good correlation with standard methods for *in vivo* proliferation analysis like thymidine analog labeling (Maslov et al., 2004) with the advantage of being independent of cell cycle length which may alternate during development and stem cell population (Calegari and Huttner, 2003; Calegari et al., 2005). MCM2 is expressed in adult as well as in embryonal neural stem cells *in vivo* (Berg et al., 2019). Together, MCM2 is—although not often used—a well-characterized and established marker for proliferation analysis in brain development (von Bohlen und Halbach, 2011).

A large body of research during the late 1980s and 1990s explored the development of the two major catecholaminergic neurotransmitter systems in the mammalian brain. All these studies looked at these systems as developing neurotransmitter systems, but could not focus on the regulation of neurogenesis or brain development, since the knowledge of such regulatory functions of neurotransmitters was rather limited at that time. Consequently, dopaminergic innervation was studied mainly in the nigrostriatal and mesolimbic systems, and noradrenergic innervation was investigated mainly preferentially in cortical areas and the hypothalamus (Coyle and Molliver, 1977; Levitt and Moore, 1979; Berger and Verney, 1984), but no data on catecholaminergic innervation of the VZ/SVZ as the main proliferating zone of the developing brain are available. Even though the two systems develop in parallel with increasing innervation of the VZ/SVZ in perinatal stages (between E16.5 and P0), we found different innervation patterns of the periventricular regions mainly containing the VZ/SVZ by the two catecholamines largely reaching the adult patterns at birth (P0): While the dopaminergic innervation focusses on the VZ/SVZ of the LV_lateral_ close to the striatum, the noradrenergic innervation omits the striatum and the adjacent periventricular region but instead reaches the midbrain/hindbrain periventricular regions. These results closely fit data from previous studies showing noradrenergic innervation in the diencephalon surrounding the 3rd ventricle and dopaminergic innervation in the striatum adjacent to the LV_lateral_ between E13 and E15 (Olson and Seiger, 1972; Ribary et al., 1986; Tomasini et al., 1997). Of note, although the general patterns of catecholaminergic innervation levels match the adult situation already in the early postnatal period, brain catecholamine levels are much higher in the adult brain compared to perinatal stages due to further postnatal maturation of the two catecholaminergic systems (Berger and Verney, 1984; Ribary et al., 1986; Tomasini et al., 1997; Murrin et al., 2007).

The major catecholamines DA and NE are pleiotropic molecules and do not only play a pivotal role in synaptic neurotransmission but are also involved in brain maturation (Felten et al., 1982; Gustafson and Moore, 1987; Berger-Sweeney and Hohmann, 1997). Particularly NE is expressed during early embryonic development and regulates both the development of the respective neuronal population and the development of its various target areas including the cortex (Berger-Sweeney and Hohmann, 1997). Consistently, the NE system regulates the development of the Cajal-Retzius cells as the first cortical neurons and is thus considered to be involved in neuronal migration and laminar formation most likely *via* α2 adrenergic receptor signaling (Wang and Lidow, 1997; Naqui et al., 1999). All these studies applied lesions of the NE system during the early postnatal period and mainly addressed cortical development and in some cases also cerebellar development (for review see Berger-Sweeney and Hohmann, 1997; Saboory et al., 2020). A single study reporting the effects of severe prenatal lesioning of the NE system by the intrauterine 6-hydroxydopamine application at E17 revealed no major alterations in cortical morphology in adulthood but did not study other brain regions (Lidov and Molliver, 1982). Our data provide first morphological evidence to support putative pleiotropic effects of NE even during earlier stages of brain development mainly in the diencephalic region as one region with high periventricular NE innervation: we detected an inverse strong negative correlation of the proliferation capacity in the periventricular neurogenic regions with their NE levels (*r* = −0.932 for log-transformed counts of proliferating cells; *P* < 0.001), but not their DA levels. Though, a functionally relevant inhibitory effect of NE on stem cell proliferation within the periventricular regions during development still needs to be confirmed by future pharmacological and genetic studies. are warranted to show the functional importance of these findings, our data suggest potent inhibitory effects of NE on stem cell proliferation within the periventricular regions during development. Consistently, prenatal treatment of rats with β-adrenoceptor agonists at gestational days 17–20 results in decreased cell numbers in the fetal brain through cAMP production in target cells (Garofolo et al., 2003; Slotkin et al., 2003). This sensitive developmental stage closely corresponds to parallels the stage of increasing NE levels and decreasing cell proliferation within the mouse VZ/SVZ at E16.5 to P0. Unfortunately, there are no detailed brain morphology studies in genetic NE depleted mice (DbH knockout mice) available, most likely due to the almost 100% early postnatal mortality without indirect NE substitution during development (Thomas et al., 1995; Jin et al., 2004). The lethal phenotype without NE supplementation during crucial developmental stages however makes it impossible to accurately study embryonic/fetal brain development under NE depletion in this model. Nevertheless, these data in the fetal brain are in strong agreement with the inhibitory effects of NE on neural stem/progenitor cell proliferation within the periventricular regions of the adult mouse brain most likely also mediated through direct stimulation of β-adrenoceptors (Weselek et al., 2020).

Together, the levels of the two major catecholamines DA and NE dramatically increased within the VZ/SVZ area of the developing brain between E16.5 and P0 with DA levels increasing in forebrain VZ/SVZs bordering the LV_lateral_ and NE levels raising in midbrain/hindbrain VZ/SVZs bordering the third ventricle, the aqueduct, and the fourth ventricle. Conversely, the number of proliferating cells of the VZ/SVZ areas dropped between E16.5 and E19.5 with a special focus on all VZ/SVZs outside the lateral ventricles leading to an inverse strong negative correlation of the proliferation capacity in the periventricular neurogenic regions with their NE levels. These results are backed by our recent findings of direct negative effects of NE on the proliferation of adult periventricular neural stem/progenitor cells (Weselek et al., 2020). Our data provide the first framework to further study the potential functional importance of catecholamines—particularly NE—in regulating neural stem and progenitor cell proliferation and differentiation during mammalian brain development.

## Data Availability Statement

The raw data supporting the conclusions of this article will be made available by the authors, without undue reservation.

## Ethics Statement

The animal study was reviewed and approved by Animal Welfare Committee at the Technische Universität Dresden and Landesdirektion Sachsen (governmental authorities).

## Author Contributions

GW, AH and AS substantially contributed to the study concept and design, acquisition of data, analyzing of data, interpretation of data, drafting and finalizing the manuscript. MG and CH substantially contributed to the acquisition of data, analyzing of data and interpretation of data. MF and FM substantially contributed to the interpretation of data, drafting and finalizing the manuscript. All authors critically revised the final manuscript.

## Conflict of Interest

The authors declare that the research was conducted in the absence of any commercial or financial relationships that could be construed as a potential conflict of interest.
