# Supplementary material for: Catecholaminergic Innervation of Periventricular Neurogenic Regions of the Developing Mouse Brain
Source: Front Neuroanat. 2020 Sep 23;14:558435. doi: 10.3389/fnana.2020.558435 (PMC7538673; doi:10.3389/fnana.2020.558435)
Supplement: Supplementary file 1 [file Data_Sheet_1.docx]

Supplementary Material

# Supplementary Figures


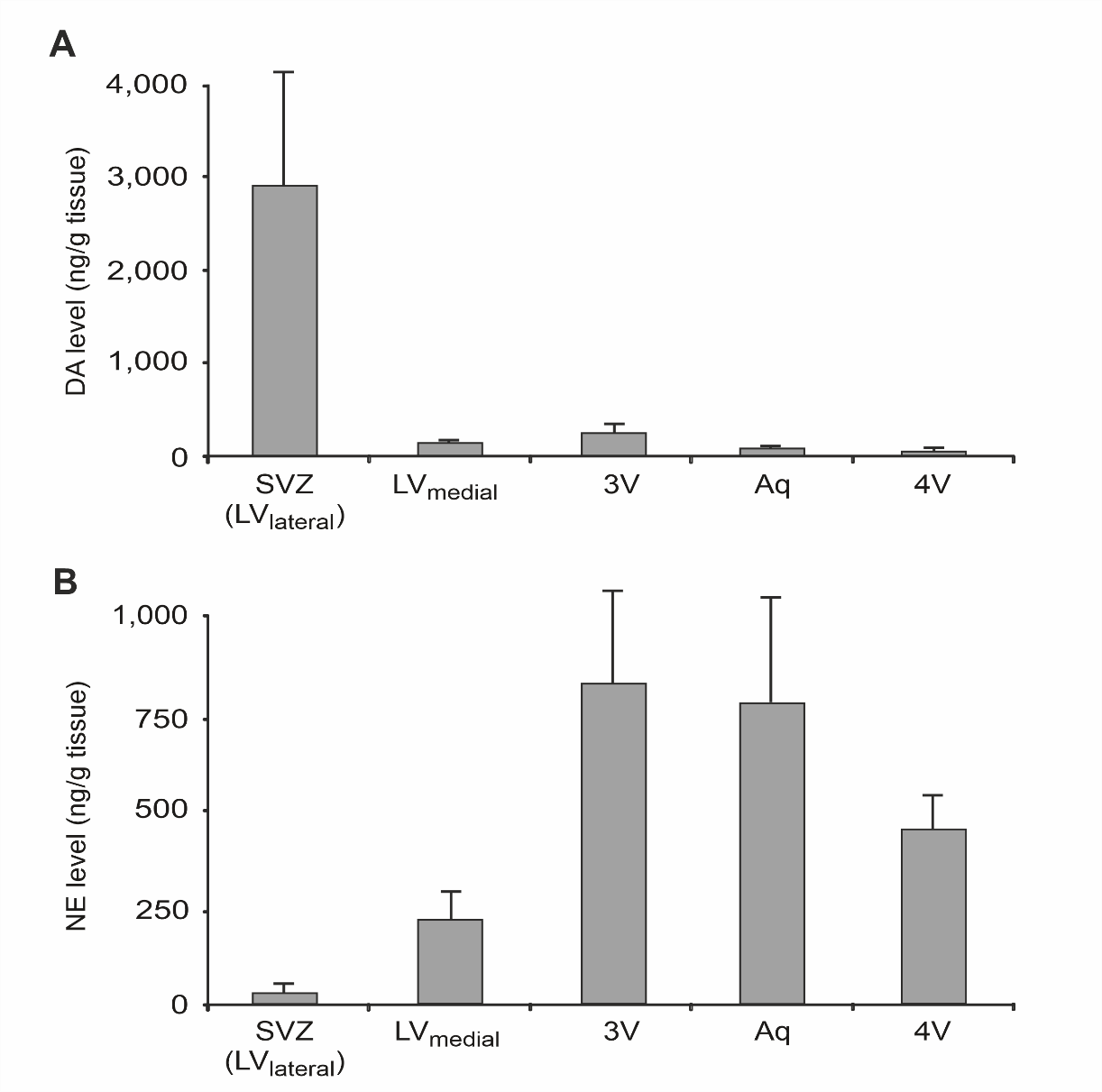


**Supplementary Figure S1:** Catecholamine levels in microdissected periventricular regions of the adult mouse brain (8 to 12 weeks). Catecholamine levels were measured in microdissected periventricular regions using a HPLC-based method and normalized to tissue weight. **(A)** Dopamine (DA) levels were high in the subventricular zone (SVZ) of the lateral walls of the lateral ventricles (LV_lateral_), but low in medial wall of the LV (LV_medial_) as well as the caudal periventricular regions bordering the 3^rd^ ventricle (3V), the aqueduct and the 4^th^ ventricle (4V) (n=4). **(B)** In contrast to DA, norepinephrine (NE) showed high levels only in the caudal (midbrain/hindbrain) periventricular regions (n=3-4). Data on NE levels from SVZ, 3V and Aq are from Weselek and co-workers (Weselek, Keiner et al. [2020]. Stem Cells, doi: 10.1002/stem.3232).

# Supplementary Tables

**Supplementary Table S1A,B:** Statistics determined for the amount of proliferative cells within the VZ (MCM2^+^ nuclei per 100µm^2^) in the different regions and developmental stages (**Figure 1**). Two-way ANOVA with *post-hoc* t-test and Bonferroni adjustment with VZ regions and developmental stages as fixed factors revealed that VZ regions and stages had a significant interaction effect on MCM2^+^ cells per 100 µm^2^ (*P*<0.001, F-value=5.7) and significant differences among VZ regions (*P*<0.001, F-value=17.1) and stages (*P*<0.001, F-value=95.0). Displayed are the Bonferroni-adjusted *P*-values. **(A)** Significances among the different VZ regions. **(B)** Significances among the developmental stages. Bold values indicate significant differences.

**A**

|  | **E14.5** | **E16.5** | **E19.5** | **P0** |
| --- | --- | --- | --- | --- |
| **LV lateral wall vs. LV medial wall** | 1.000 | 0.116 | 0.069 | 0.054 |
| **LV lateral wall vs. 3V** | **0.004** | **<0.001** | **0.043** | **0.049** |
| **LV lateral wall vs. Aqueduct** | 0.124 | **<0.001** | **0.025** | **0.017** |
| **LV lateral wall vs. 4V** | 1.000 | **<0.001** | **0.036** | **0.046** |
| **LV medial wall vs. 3V** | **<0.001** | **0.025** | 1.000 | 1.000 |
| **LV medial wall vs. Aqueduct** | 0.546 | **0.009** | 1.000 | 1.000 |
| **LV medial wall vs. 4V** | 1.000 | **0.017** | 1.000 | 1.000 |
| **3V vs. Aqueduct** | **<0.001** | 1.000 | 1.000 | 1.000 |
| **3V vs. 4V** | **0.001** | 1.000 | 1.000 | 1.000 |
| **Aqueduct vs. 4V** | 0.956 | 1.000 | 1.000 | 1.000 |

**B**

|  | **LV lateral wall** | **LV medial wall** | **3V** | **Aqueduct** | **4V** |
| --- | --- | --- | --- | --- | --- |
| **E14.5 vs. E16.5** | **0.043** | 1.000 | 1.000 | **<0.001** | **0.001** |
| **E14.5 vs. E19.5** | **0.003** | **<0.001** | **0.046** | **<0.001** | **<0.001** |
| **E14.5 vs. P0** | **<0.001** | **<0.001** | **0.025** | **<0.001** | **<0.001** |
| **E16.5 vs. E19.5** | **<0.001** | **<0.001** | 0.122 | 0.148 | **0.022** |
| **E16.5 vs. P0** | **<0.001** | **<0.001** | 0.074 | 0.123 | **0.017** |
| **E19.5 vs. P0** | 1.000 | 1.000 | 1.000 | 1.000 | 1.000 |

**Supplementary Table S2A,B:** Statistics determined for the DA levels within the VZ in the different regions and developmental stages (**Figure 3A**). Two-way ANOVA with *post-hoc* t-test and Bonferroni adjustment with VZ regions and developmental stages as fixed factors revealed that VZ regions and stages had a significant interaction effect on DA levels (*P*<0.001, F-value=13.3) and significant differences among VZ regions (*P*<0.001, F-value=10.3) and stages (*P*<0.001, F-value=14.1). Displayed are the Bonferroni-adjusted *P*-values. **(A)** Significances among the different VZ regions. **(B)** Significances among the developmental stages. Bold values indicate significant differences.

**A**

|  | **E14** | **E16** | **P0** |
| --- | --- | --- | --- |
| **LV lateral wall vs. LV medial wall** | 1.000 | 1.000 | **<0.001** |
| **LV lateral wall vs. 3V** | **0.049** | 1.000 | **<0.001** |
| **LV lateral wall vs. Aqueduct** | 1.000 | 1.000 | **<0.001** |
| **LV lateral wall vs. 4V** | 1.000 | 1.000 | **<0.001** |
| **LV medial wall vs. 3V** | **0.028** | 0.731 | 0.415 |
| **LV medial wall vs. Aqueduct** | 0.724 | 1.000 | 1.000 |
| **LV medial wall vs. 4V** | 1.000 | 0.699 | 1.000 |
| **3V vs. Aqueduct** | 1.000 | 1.000 | 1.000 |
| **3V vs. 4V** | 0.211 | 1.000 | **0.028** |
| **Aqueduct vs. 4V** | 1.000 | 1.000 | 0.412 |

**B**

|  | **LV lateral wall** | **LV medial wall** | **3V** | **Aqueduct** | **4V** |
| --- | --- | --- | --- | --- | --- |
| **E14 vs. E16** | 0.239 | 0.256 | **0.010** | **0.031** | 1.000 |
| **E14 vs. P0** | **<0.001** | **0.038** | **0.001** | **0.001** | **<0.001** |
| **E16 vs. P0** | **<0.001** | 1.000 | 1.000 | 1.000 | **0.002** |

**Supplementary Table S3A,B:** Statistics determined for the NE levels within the VZ in the different regions and developmental stages (**Figure 3B**). Two-way ANOVA with *post-hoc* t-test and Bonferroni adjustment with VZ regions and developmental stages as fixed factors revealed that VZ regions and stages had a significant interaction effect on NE levels (*P*=0.001, F-value=4.3) and significant differences among VZ regions (*P*=0.003, F-value=5.3) and stages (*P*<0.001, F-value=32.4). Displayed are the Bonferroni-adjusted *P*-values. **(A)** Significances among the different VZ regions. **(B)** Significances among developmental stages. Bold values indicate significant differences.

**A**

|  | **E14** | **E16** | **P0** |
| --- | --- | --- | --- |
| **LV lateral wall vs. LV medial wall** | 1.000 | 1.000 | 1.000 |
| **LV lateral wall vs. 3V** | 1.000 | 1.000 | 0.134 |
| **LV lateral wall vs. Aqueduct** | 1.000 | 1.000 | **<0.001** |
| **LV lateral wall vs. 4V** | 1.000 | 0.418 | **0.003** |
| **LV medial wall vs. 3V** | 1.000 | 1.000 | 1.000 |
| **LV medial wall vs. Aqueduct** | 1.000 | 1.000 | **0.002** |
| **LV medial wall vs. 4V** | 1.000 | 0.516 | 0.531 |
| **3V vs. Aqueduct** | 1.000 | 1.000 | **0.006** |
| **3V vs. 4V** | 1.000 | 1.000 | 1.000 |
| **Aqueduct vs. 4V** | 1.000 | 0.374 | 0.185 |

**B**

|  | **LV lateral wall** | **LV medial wall** | **3V** | **Aqueduct** | **4V** |
| --- | --- | --- | --- | --- | --- |
| **E14 vs. E16** | 1.000 | 1.000 | 1.000 | 1.000 | **0.036** |
| **E14 vs. P0** | 1.000 | 0.067 | 0.110 | **<0.001** | **<0.001** |
| **E16 vs. P0** | 1.000 | 0.256 | 0.265 | < 0.001 | 0.263 |
